# Extracellular Vesicles Derived-LAT1 mRNA as a Powerful Inducer of Colorectal Cancer Aggressive Phenotype

**DOI:** 10.3390/biology11010145

**Published:** 2022-01-15

**Authors:** Cristina Almeida, Ana Luísa Teixeira, Francisca Dias, Vera Machado, Mariana Morais, Gabriela Martins, Carlos Palmeira, Maria Emília Sousa, Inês Godinho, Sílvia Batista, Bruno Costa-Silva, Rui Medeiros

**Affiliations:** 1 Molecular Oncology and Viral Pathology Group, Research Center of IPO Porto (CI-IPOP)/RISE@CI-IPOP (Health Research Network), Portuguese Oncology Institute of Porto (IPO Porto)/Porto Comprehensive Cancer Center (Porto.CCC), Rua Dr. António Bernardino de Almeida, 4200-072 Porto, Portugal; cristina.almeida@ipoporto.min-saude.pt (C.A.); francisca.carvalho.dias@ipoporto.min-saude.pt (F.D.); vera.pereira.machado@ipoporto.min-saude.pt (V.M.); mariana.gomes.morais@ipoporto.min-saude.pt (M.M.); ruimedei@ipoporto.min-saude.pt (R.M.); 2 Research Department of the Portuguese League against Cancer Regional Nucleus of the North (LPCC-NRN), Estrada da Circunvalação 6657, 4200-177 Porto, Portugal; 3 ICBAS School of Medicine and Biomedical Sciences, University of Porto (UP), Rua Jorge Viterbo Ferreira 228, 4050-513 Porto, Portugal; 4 Immunology Department, Portuguese Oncology Institute of Porto (IPO Porto), Rua Dr. António Bernardino de Almeida, 4200-072 Porto, Portugal; gmartins@ipoporto.min-saude.pt (G.M.); carlospalmeira@ipoporto.min-saude.pt (C.P.); emilia.sousa@ipoporto.min-saude.pt (M.E.S.); ines.godinho@ipoporto.min-saude.pt (I.G.); 5 Pathology and Experimental Therapeutic Group, Research Center of IPO Porto (CI-IPOP)/RISE@CI-IPOP (Health Research Network), Portuguese Oncology Institute of Porto (IPO Porto)/Porto Comprehensive Cancer Center (Porto.CCC), Rua Dr. António Bernardino de Almeida, 4200-072 Porto, Portugal; 6 Fernando Pessoa Research, Innovation and Development Institute (I3ID FFP), Fernando Pessoa University (UFP), Praça 9 de Abril 349, 4249-004 Porto, Portugal; 7 Systems Oncology Group, Champalimaud Research, Champalimaud Centre for the Unknown, Av. Brasília, 1400-038 Lisbon, Portugal; silvia.batista@research.fchampalimaud.org (S.B.); bruno.costadasilva@research.fchampalimaud.org (B.C.-S.); 8 Faculty of Medicine, University of Porto (FMUP), Alameda Prof. Hernâni Monteiro, 4200-319 Porto, Portugal

**Keywords:** colorectal cancer (CRC), mRNAs, LAT1, ASCT2, extracellular vesicles (EV’s)

## Abstract

**Simple Summary:**

The metastatic spread of tumor cells to the liver is one of the most common causes of mortality in CRC. Extracellular vesicles are currently considered vehicles of metastization, playing a role in the modification of the recipient cell’s phenotype. LAT1 and ASCT2 are amino acids transporters associated with increased proliferation in CRC. This study analyzed the effect of *LAT1* and *ASCT2* mRNAs derived from CRC-EVs in the phenotype modulation of recipient cells. In this study we demonstrate *LAT1*-EVs mRNA involvement in recipient cells’ phenotype modulation, conferring advantages in cell migration and proliferation.

**Abstract:**

Colorectal cancer (CRC) is the third most common cancer in the world and represents the third most deadly tumor worldwide. About 15–25% of patients present metastasis in the moment of diagnosis, the liver being the most common site of metastization. Therefore, the development of new therapeutic agents is needed, to improve the patients’ prognosis. Amino acids transporters, LAT1 and ASCT2, are described as upregulated in CRC, being associated with a poor prognosis. Extracellular vesicles have emerged as key players in cell-to-cell communication due to their ability to transfer biomolecules between cells, with a phenotypic impact on the recipient cells. Thus, this study analyzes the presence of *LAT1* and *ASCT2* mRNAs in CRC-EVs and evaluates their role in phenotype modulation in a panel of four recipient cell lines (HCA-7, HEPG-2, SK-HEP-1, HKC-8). We found that HCT 116-EVs carry *LAT1*, *ASCT2* and other oncogenic mRNAs being taken up by recipient cells. Moreover, the HCT 116-EVs’ internalization was associated with the increase of *LAT1* mRNA in SK-HEP-1 cells. We also observed that HCT 116-EVs induce a higher cell migration capacity and proliferation of SK-HEP-1 and HKC-8 cells. The present study supports the *LAT1*-EVs’ mRNA involvement in cell phenotype modulation, conferring advantages in cell migration and proliferation.

## 1. Introduction

Colorectal cancer (CRC) represents 10% of all diagnosed cancers, being the third most frequent cancer worldwide [1]. According to World Health Organization (WHO), CRC is also the third most deadly cancer [2]. In fact, patients that are diagnosed at advanced stages of CRC present 5-year survival rates of 12%, compared to the 90% observed in patients diagnosed at early stages of the disease [3]. However, approximately 15–25% of CRC patients present a metastatic disease at diagnosis, with an increase to 50% during the course of the disease. The liver is the most frequent initial site of metastases establishment, and although extremally rare, kidney metastasis formation can also occur [4,5,6,7]. Therefore, the definitions of new prognosis, follow-up molecular biomarkers and therapeutic agents are imperative to improve CRC patients’ care and promote a more individualized and precise follow-up.

In 2011, metabolic reprogramming was defined as a cancer “hallmark”, being essential to support tumor cells’ growth and progression in response to altered microenvironments [8]. In fact, cancer cells present plasticity and adaptative capacity in response to microenvironment alterations to continue to proliferate and adapt to dynamic changes, the amino acid (AA) bioavailability being an important necessity to answer the high demand of nutrients during the cancer progression [9]. Thus, solute carriers’ transporters (SLCs) present a key role in CRC progression, since these transmembrane carriers are involved in the transport of several solutes such as AA, lipids and inorganic solutes, participating in key carcinogenesis processes, such as proliferation, apoptosis, invasion and metastases formation [10,11]. 

Recently, the overexpression of some AA transporters was associated with the increased AA demand of cancer cells [12]. LAT1 and ASCT2 are two of the SLCs deregulated during CRC progression [10,13,14]. LAT1 is a sodium (Na^+^) and pH independent transporter, involved in the essential amino acids’ (EAAs) transport [14,15,16]. It forms a heterodimer complex with CD98 to import large and neutral AAs, in exchange for the efflux of intracellular substrates, including EAAs and glutamine [12,15,16,17]. On the other hand, ASCT2 is a Na^+^ and pH dependent transporter, responsible for the transport of glutamine inside of cells [18,19,20,21]. The overexpression of LAT1 and ASCT2 is related with patients’ poor prognosis [18,22,23,24]. Furthermore, the upregulation of LAT1 is frequently observed in CRC, liver and kidney cancers [15,16,22,24,25,26], and its knockdown has been associated with the reduction of leucine uptake and cell proliferation [16]. ASCT2 overexpression has also been reported in several cancers, including breast, lung cancer and hepatocellular carcinoma, and its expression levels were also associated with tumor depth and the vascular invasion in KRAS-mutant CRC [27,28,29,30,31]. Interestingly, Toda and co-workers observed that ASCT2-knockdown presents a more suppressive effect on cell growth than glutamine depletion [27]. Namikawa and co-workers showed that the overexpression of both transporters in hepatocellular carcinoma was associated with metastases development and disease aggressiveness [32]. 

Extracellular vesicles (EV’s) have been recognized as key mediators of cell communication and microenvironment modulation. They can transfer molecular information (such as DNA, RNA and protein) between cells and modify their phenotype [33]. EVs are small nanovesicles surrounded by a membrane composed by a lipid bilayer and hydrophilic proteins, being released by numerous cell types, both in physiologic and pathologic conditions [34,35,36,37,38]. EVs play an important role in the pre-metastatic niche establishment, promoting the upregulation of inflammatory molecules, immune suppression, increasing angiogenesis and vascular permeability, and determining organotropism metastases [39]. In fact, Chiba and co-workers have reported that the EVs secreted from human CRC cells can transfer RNAs to liver cells, and modify their phenotype features, inducing invasion, intravasation and metastatic capacity [40]. Thus, considering the EVs’ ability to modify the phenotype of recipient cells, our aim was to analyze the effect of *LAT1* and *ASCT2* mRNA derived CRC-cells’ EVs on the phenotype modulation of recipient cells to clarify their potential tropism.

## 2. Materials and Methods

### 2.1. Cell Culture

A panel of five cell lines was used: HCT 116 and HCA-7 (CRC cell lines), SK-HEP-1 and HEPG-2 (hepatocellular carcinoma cell lines) and HKC-8 (human-derived normal renal proximal epithelial tubular cell line). HCT 116 and SK-HEP-1 cells were kindly provided by the Biomedicine Department of Faculty of Medicine of Porto University and HKC-8 was obtained from the Department of Genetics of University Medical Center of Groningen. HEPG-2 cell line was provided by the Basic and Clinical Research on Iron Biology Group from I3S. HCA-7 was obtained from American Type Culture Collection (ATCC^®^ RRID: CVCL_0289). Briefly, HCT 116 cells were maintained in McCoy’s 5a medium (Sigma-Aldrich^®^, St. Louis, MO, USA), supplemented with 2.2 g/L sodium bicarbonate (Merck^®^, Darmstadt, Germany), 6 g/L Hepes (Sigma-Aldrich^®^), 10% fetal bovine serum (FBS) (Gibco-Thermo Fisher Scientific^®^, Waltham, MA, USA) and 1% of pen-strep (penicillium-streptomycin mixture) (Gibco^®^, Thermo Fisher Scientific^®^). The SK-HEP-1 cells were maintained in RPMI medium (Sigma-Aldrich^®^), with 2.2. g/L sodium bicarbonate (Merck^®^), 6 g/L Hepes (Sigma-Aldrich^®^), 10% FBS (Gibco^®^, Thermo Fisher Scientific^®^) and 1% of pen-strep (Gibco^®^, Thermo Fisher Scientific^®^). The HCA-7 and HEPG-2 cells were maintained in DMEM medium (Gibco^®^, Thermo Fisher Scientific^®^), supplemented with 10% of FBS (Gibco^®^, Thermo Fisher Scientific^®^) and 1% of pen-strep (Gibco^®^, Thermo Fisher Scientific^®^). Additionally, HKC-8 cells were maintained in DMEM/F12 medium supplemented with ITS (Insulin-Transferrine-Selenum) (Sigma-Aldrich^®^), EGF (Epidermal Growth Factor) (Sigma-Aldrich^®^), Hepes buffer (Sigma-Aldrich^®^), pen-strep (Gibco^®^, Thermo Fisher Scientific^®^) and hydrocortisone (Sigma-Aldrich^®^). The cell lines were kept in an incubator at 37 °C with 5% of CO_2_. All cells were routinely tested for mycoplasma presence every two weeks, being free from contamination. 

### 2.2. EVs’ Isolation

The HCT 116 cell line was used as EVs’ producer. Briefly, the HCT 116 cell line was cultured in the normal medium until it reached 80–90% of confluence. The medium was then replaced by McCoy’s 5A supplemented with 10% of exosome-depleted (exo-free) FBS (Thermo Fisher Scientific^®^) (McCoy’s Exo-free) for 48 h. Then, the cell culture medium was harvested and centrifuged for 30 min at 3500 rpm at 4 °C. Subsequently, the supernatant was filtered with a 0.22 µm filter (GE Healthcare Whatman^TM^, Chicago, IL, USA). The purified supernatant and the EV’s isolation reagent (Total Exosome Isolation Reagent) (Thermo Fisher Scientific^®^) were mixed in a 2:1 proportion and incubated overnight at 4 °C. The mixture was then centrifuged at 10,000× *g*, at 4 °C, for 1 h and the pellet (EVs’ fraction) was resuspended in filtered PBS (Gibco^®^, Thermo Fisher Scientific^®^). 

### 2.3. EVs’ Characterization

The HCT 116-EVs were analyzed for size distribution by the NS300 Nanoparticle Tracking Analysis (NTA) system (NanoSight—Malvern Panalytical, Malvern, UK). Samples were pre-diluted in filtered PBS 1X (Gibco^®^, Thermo Fisher Scientific^®^) to achieve a concentration within the range for optimal NTA analysis. Video acquisitions were made using a camera level of 16 and a threshold between 5 and 7. Five to nine videos of 30 s were captured per sample. Analysis of particle size distribution was performed with NTA software v3.4 (Figure 2A). EVs’ quantification in our isolates was made using a CFSE (Carboxyfluorescein succinimidyl ester) (ab113853—Abcam^®^, Cambridge, UK) staining protocol, optimized for a conventional flow cytometer, using a BD FACS Canto II flow cytometer (BD Biosciences, Franklin Lakes, NJ, USA). Before acquisition, an intensive distilled water cleaning of the fluidic system of the cytometer was performed. We defined the cytometer settings for EVs’ analysis using Megamix-Plus SSC beads (BioCytex, Marseille, France), a mix of fluorescent beads of different diameters that cover a variety of microparticle size ranges (0.16 to 0.40 μm) (Figure 2B) and use side scatter (SSC) as a size-related parameter. The EVs’ suspension was stained with CFSE at a final concentration of 2 μM and incubated in the dark at 37 °C for 45 min. Finally, EVs’ morphology and shape were analyzed by Transmission Electron Microscopy (TEM) (Figure 2E).

### 2.4. Quantitative Real-Time Polymerase Chain Reaction (qRT-PCR)

The mRNA from all the cell lines and HCT 116-EVs was isolated using the GRS Total RNA Kit- Blood & Cultured Cells (Gibco^®^, Thermo Fisher Scientific^®^), according to the manufacturer’s protocol. When the cells reached a confluence of 80–90%, the culture medium was collected for EVs’ isolation and, subsequently, 3 × 10^6^ cells were used for mRNA extraction. For each condition used in this study, the procedure was replicated three times. The mRNA samples were used as templates for complementary cDNA synthesis, using the High-Capacity cDNA Reverse Transcription Kit (Applied Biosystems^®^, Thermo Fisher Scientific^®^, Waltham, MA, USA), according to the manufacturer’s protocol. The mRNA expression levels were analyzed by Real Time PCR and the reactions were carried out on a StepOne^TM^qPCR Real-Time PCR machine, containing 1X Taqman^TM^ Fast advanced master mix (Applied Biosystems^®^, Thermo Fisher Scientific^®^), with 1X probes (TaqMan^®^mRNA Expression Assays: *LAT1* (Hs01001186_m1), *ASCT2* (Hs01056542_m1), *HIF1-A* (Hs00153153_m1), *EGFR* (Hs01076078_m1), VEGFA (Hs00900055_m1), *CXCR4* (Hs00607978_s1) and cDNA sample (≈50 ng). *B2M* (Beta-2-Macroglobulin (Hs99999907_m1)) was used as endogenous control for mRNA data normalization and data analysis was made using StepOneTM Sofware v2.2 (Applied Biosystems^®^, Thermo Fisher Scientific^®^). All quantification reactions were performed in duplicate and negative controls were included in each run. 

### 2.5. EVs’ Uptake Studies

The internalization of HCT 116-EVs by the recipient cells (HCA-7, SK-HEP-1, HEPG-2 and HKC-8) was analyzed through fluorescence analysis. Additionally, their impact in the recipient cells was analyzed in terms of cell proliferation, migration capacity, transcription, and protein levels.

Thus, 2 × 10^5^ cells of recipient cells were seeded in a 6 multi-well plate and maintained, according to the conditions previously described. When the cells reached 60–70% of confluence, the medium was changed to exo-free medium and the HCT 116-EVs were administrated in two different concentrations: (1) 5.3 × 10^8^ EVs/mL (HCT 116-EVs condition 1) and 15.9 × 10^8^ EVs/mL (HCT 116-EVs condition 2).

#### 2.5.1. EVs’ Uptake by Recipient Cells 

Firstly, the HCT 116-EVs were labeled with 0.5 µL of CFSE membrane-permeable fluorescent dye (ab113853—Abcam^®^), for 45 min, in the dark, at 37 ºC. Then, the labeled HCT 116-EVs’ conditions 1 and 2 were resuspended in fresh culture medium and added to the HCA-7, SK-HEP-1, HEPG-2 and HKC-8 cell lines which were cultured in cover slips in 6 multi-well plates, for 24 h. Subsequently, each well was washed with PBS 1X (Gibco^®^, Thermo Fisher Scientific^®^), three times, and the cells were incubated with 4% paraformaldehyde for 15 min, followed by three washes with PBS 1X (Gibco^®^, Thermo Fisher Scientific^®^). Then, 1 mL of DAPI (4′,6′-diamino-2-fenil-indol) (Thermo Fisher Scientific^®^) was added to each well for 5 min to stain the cell’s nucleus. Finally, Kaiser’s glycerol gelatin (Merck^®^) was added to the cover slip and then preparations were analyzed using an Olympus^®^ IX51 microscope. 

#### 2.5.2. Cell Phenotypic Assays 

To evaluate the cell migration capacity, we performed a wound healing assay. Briefly, 2 × 10^6^ cells of recipient cells were cultured, in a 6-multi well plate and, after 24 h, a scratch was performed in the confluent cell monolayer and the HCT 116-EVs’ conditions 1 and 2 were added with 1.5 mL of exo-free medium. The scratch distances and wound closure was evaluated using the beWound software (beWound-Cell Migration tool (Version 1.5)) [41]. The relative migration distances were calculated according to the following formula: % of wound closure = 100 × (d_0_ − d_t_)/mean of d_0_, where d_0_ is the width of cell wounds at time point 0 h, and d_t_ is the width of cell wounds at different time points. Additionally, in the moment of scratch closure, a proliferation assay was made. To analyze cell proliferation, 2 × 10^5^ recipient cells were plated per well in a 96 multi-well and, after 24 h, the HCT 116-EVs were administrated in the two concentrations (5.3 × 10^8^ EVs/mL and 15.9 × 10^8^ EVs/mL), as described above. After 24 h of incubation, the WST-1 reagent (Abcam^®^, Cambridge, United King) was added and incubated for 1 h and the absorbance was read at 450 nm. 

#### 2.5.3. EVs’ Impact on Transcriptional Profile of Recipient Cells

To analyze the effect of EVs’ mRNA cargo in the recipient cells, 2 × 10^6^ cells (of each of the recipient cells analyzed) were seeded in a 6 multi-well plate and, after 24 h, the medium was replaced by exo-free medium and the HCT 116-EVs’ conditions 1 and 2 were administrated. Following 24 h, the cells were trypsinized and the total of 7 × 10^6^ cells of each cell line were recovered for mRNA extraction. The protocol used for mRNA extraction, quantification, and analysis was as previously described in Section 2.4. 

#### 2.5.4. LAT1 and ASCT2 Protein Levels’ Analysis

To quantify the protein levels of LAT1 and ASCT2, we performed a Western blot assay. For protein extraction, 2 × 10^6^ cells were cultured in the respective culture medium. When cells reached a confluence of 80–90%, they were trypsinized and centrifuged for 10 min at 2500× *g*, at 4 °C. The pellet was then lysed with 150 µL of RIPA buffer (Radioimmunoprecipitation Assay Buffer) (Santa Cruz Biotechnology^®^, Dallas, TX, USA) and supplemented with 1.5 µL of phosphatase inhibitor cocktail (Thermo Fisher Scientific^®^). Subsequently, the cell lysates were centrifugated for 15 min at 14,000× *g*, at 4 °C, and the supernatant was recovered for protein quantification using a DC Protein Assay (BioRad Laboratories^®^, Hercules, CA, USA), measuring the solution’s absorbance at 750 nm. The electrophoretic separation of proteins (20 μg) was performed in Mini-Protean TGX Gels (4–20%) (BioRad Laboratories^®^). The separated proteins were electrotransferred to polyvinylidene difluoride (PVDF) membranes (BioRad Laboratories^®^) and blocked using 5% BSA (Albumin Bovine Fraction V) (Enzytech^®^, Lisbon, Portugal) in Tris-buffered saline with Tween20 (TBS-T). Following on, the membranes were incubated with primary antibodies (LAT1 (5347S) (1:250) (Cell Signaling Technology^®^, Danvers, MA, USA), ASCT2 (8057S) (1:800) (Cell Signaling Technology^®^), GAPDH (1:800) (sc-365062) (Santa Cruz Biotechnology^®^)), overnight at 4 °C. Subsequently, the membranes were incubated with conjugated secondary antibodies (anti-mouse (sc-2005) (1:10,000) (Santa Cruz Biotechnology^®^) and anti-rabbit (7074S) (1:3000) (Cell Signaling Technology^®^)) for 1 h at room temperature. The chemiluminescence was evaluated using ECL^TM^ Prime Western Blotting System (Cytiva^TM^, Amersham, UK), according to manufacturer’s instructions. The experiment was replicated 2 times. 

### 2.6. Statistical Analysis

Statistical analysis was made using IBM^®^SPSS^®^Statisticals for Windows v23. To evaluate the statistic differences in the normalized expression levels of the mRNAs, the 2^−ΔΔCq^ method, along with the Student’s *t*-test were used [42]. Data are expressed as the mean ± standard error for each group. One-way analysis of variance was used to analyze the difference between groups and the least significance difference test was used for comparisons between two groups. *p* < 0.01 and *p* < 0.05 were considered to indicate a statistically significant difference. Additionally, GraphPad Prism 8 was used for graphical presentation of the data. 

## 3. Results

### 3.1. Cell Lines Transcriptional Profile Characterization 

**Figure 1:**
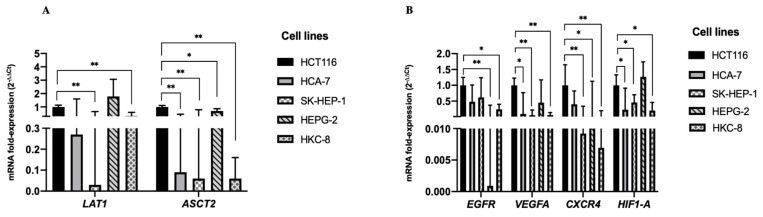
The bars represent the fold change of the mRNAs’ expression, normalized to *B2M*. Expression levels shown are means of three technical replicates for each sample. (**A**) The graphs show the increase of *LAT1* and *ASCT2* mRNA and (**B**) *EGFR*, *VEGFA*, *CXCR4* and *HIF1–A* mRNAs expression levels in HCT 116 cells, compared to the others cell lines in the analyzed panel. (Mean ± Std. Error, ** *p* < 0.001, * *p* < 0.05).

As previously mentioned, the transcriptional levels of *LAT1* and *ASCT2* mRNA were quantified by real-time qPCR in the HCT 116, SK-HEP-1, HEPG-2, HCA-7 and HKC-8 cell lines. Moreover, we also analyzed the levels of *EGFR*, *VEGFA*, *CXCR4* and *HIF1-A* mRNAs, considering their oncogenic potential role. We found higher expression levels of *LAT1* mRNA in the HCT 116 cell line compared to SK-HEP-1 (*p* < 0.001) and HKC-8 (*p* = 0.001) cell lines (Figure 1A). The HCT 116 cell line also presented higher levels of *ASCT2* mRNA compared to HCA-7 (*p* < 0.001), SK-HEP-1 (*p* < 0.001), HEPG-2 (*p* = 0.035) and HKC-8 (*p* < 0.001) (Figure 1A).

Regarding the *EGFR* mRNA levels, we also found an increase in the expression levels in the HCT 116 cell line compared to the HEPG-2 (*p* < 0.001) and HKC-8 (*p* = 0.001) cell lines. We also observed higher mRNA levels of *VEGFA*, *CXCR4* and *HIF1-A* mRNAs in the HCT 116 cell line compared to the SK-HEP-1 and HKC-8 cell lines. Concerning the *VEGF* (*p* = 0.001) and *HIF1-A* (*p* = 0.020) mRNAs, higher levels were also found in the HCT 116 compared to the other CRC cell line, HCA-7. The HEPG-2 cell line presented lower levels of *CXCR4* (*p* = 0.002) mRNA compared to the HCT 116 (Figure 1B).

### 3.2. HCT 116-EVs’ Characterization 

**Figure 2:**
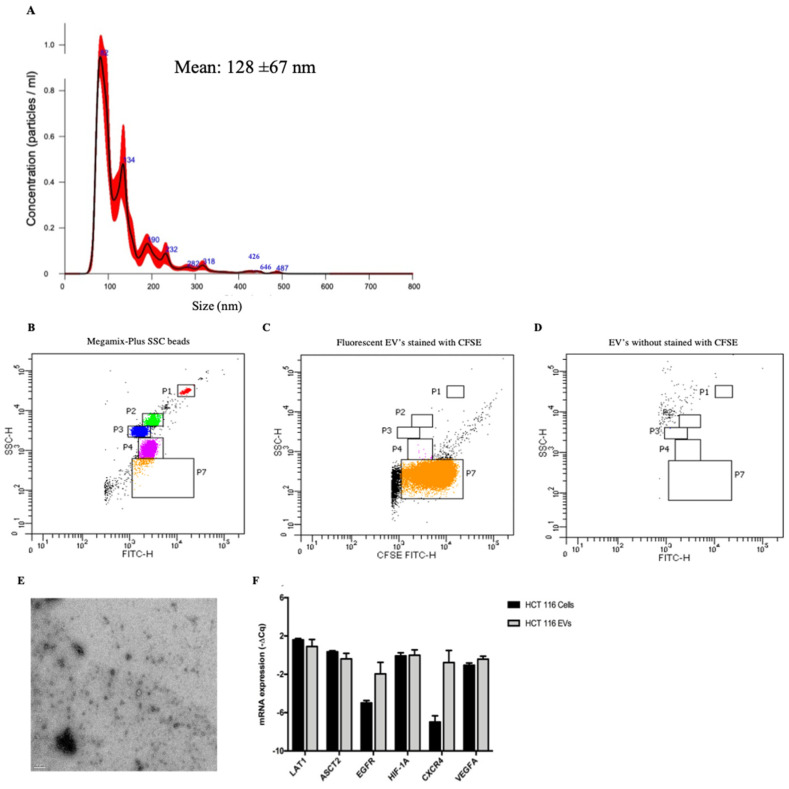
(**A**) NTA analysis of HCT 116-EVs showing the size distribution of EVs. (**B**) Megamix-Plus SSC beads used to define the cytometer settings for EVs’ acquisition, with the following diameters: 0.5 μm (gate P1), 0.24 μm (gate P2), 0.20 μm (gate P3) and 0.16 μm (gate P4); (**C**) Fluorescent EVs (approximately 2.6 × 10^5^/200 µL) as observed by flow cytometry from a sample of EVs isolate derived from HCT 116 cell line stained with CFSE (gate P7); (**D**) Negative control of EVs isolate derived from HCT 116 cell line without previous staining (gate P7). (**E**) Transmission electron microscopy (TEM) of EVs derived from HCT 116 cell line (scale 200 nm). The TEM image was acquired in the Histology and Electron Microscopy platform from I3S Porto using a Transmission Electron Microscope Jeol JEM 1400. (**F**) The bars represent the –ΔCq of the mRNAs’ expression, normalized to *B2M*. The graph shows the presence of intracellular and EVs related mRNAs levels (*LAT1*, *ASCT2*, *EGFR*, *HIF1-A*, *CXCR4* and *VEGFA)* derived from HCT 116 cell line. (Mean ± Std. Error).

The EVs were characterized according to size, shape, and quantity. The NTA analysis indicated that the vast majority of isolated EVs presented a mean size of 128 nm, which is consistent with the size of small EVs (Figure 2A). These results are consistent with the flow cytometry analysis, where were found the presence of EVs smaller than 160 nm (Figure 2B–D). The Transmission Electron Microscopy (TEM) image (Figure 2E) shows the variability of morphology presented in HCT 116-EVs. Additionally, we validated the presence of *LAT1*, *ASCT2*, *EGFR*, *HIF1-A*, *CXCR4* and *VEGFA* mRNAs in HCT 116-EVs (Figure 2F).

### 3.3. HCT 116-EVs’ Uptake Effect on HCA-7, SK-HEP-1, HEPG-2 and HKC-8 Recipient Cells 

**Figure 3:**
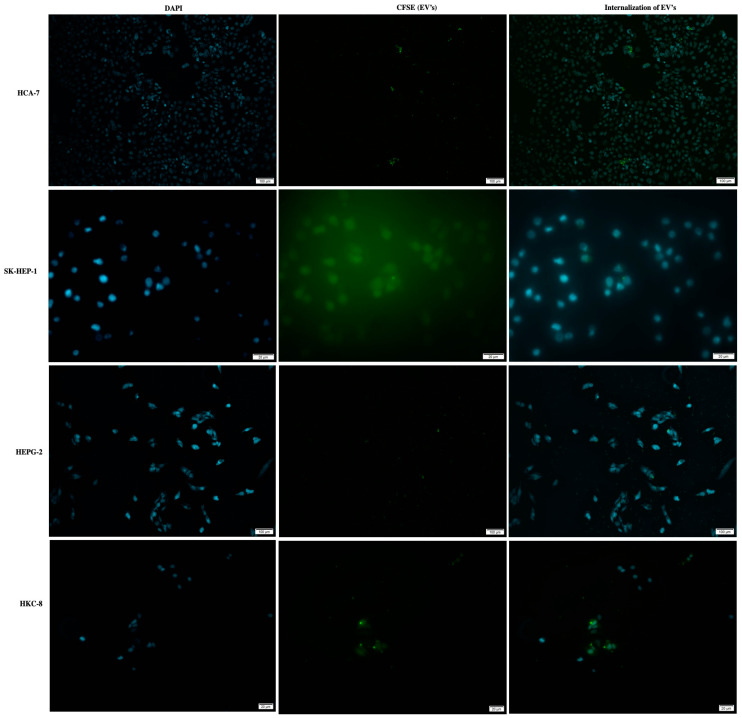
Representative immunofluorescence image shows the internalization of HCT 116-EVs labeled with CFSE (green dye) by HCA-7, SK-HEP-1, HEPG-2 and HKC-8 cells. The cell’s nucleus is stained with DAPI (blue dye). (10X Olympus^®^ IX51 microscope).

Firstly, we analyzed the internalization/uptake of HCT 116-EVs in HCA-7, HEPG-2, SK-HEP-1 and HKC-8 recipient cell lines. As we can observe in Figure 3, the CFSE labeled HCT 116-EVs were internalized by all recipient cell lines (right images of Figure 3), validating the concept of cellular communication through EVs’ networks.

**Figure 4:**
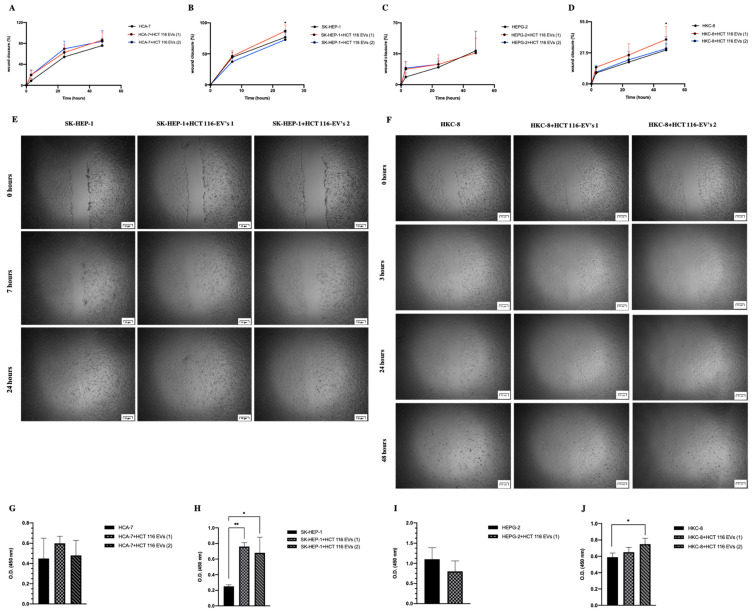
HCT 116-EVs’ uptake in HCA-7 (**A**), SK-HEP-1 (**B**), HEPG-2 (**C**) and HKC-8 (**D**), respectively, affects cell migration capacity of recipient cells, with HCT 116-EVs condition 1 being able to induce an increase in gap closure in SK-HEP-1 and HKC-8 recipient cells. Representative images of wound healing assay in (**E**) SK-HEP-1 and (**F**) HKC-8 cells with internalization of HCT 116-EVs (conditions 1 and 2). Additionally, the HTC 116-EVs’ uptake induces a higher proliferation rate in receptor cells SK-HEP-1 (**H**) and HKC-8 cells (**J**). However, this effect was not found in HCA-7 (**G**) or in HEPG-2 (**I**). The represented phenotypic assays were replicated three times for each sample. The statistical analysis was performed between the control group (recipient cells) and recipient cells after uptake of HCT 116-EVs (condition 1 and condition 2) in cells, at the same time point. (Mean ± Std. Error, ** *p* < 0.001, * *p* < 0.05). (**E**) Scale bar = 20 μm; (**F**) Scale bar = 50 μm.

We then evaluated the HCT 116-EV’s effect (condition 1: 5.3 × 10^8^ EV’s/mL and condition 2: 15.9 × 10^8^ EV’s/mL) on cell proliferation and migration (Figure 4A–D) and proliferation capacity (Figure 4G–J) of the recipient cells: HCA-7 (Figure 4A,G); SK-HEP-1 (Figure 4B,H); HEPG-2 (Figure 4C,I) and HKC-8 (Figure 4D,J). 

The figures of the wound healing assays of all recipient cells can be observed in the Supplementary Material (Figure S1). All the represented statistical analyses were performed between the control condition (recipient cells) and the uptake internalization (HCT 116-EVs 1 or HCT 116-EVs 2), at the same time point. According to the results, the uptake of HCT 116-EVs 1 by the SK-HEP-1 (*p* = 0.002) and HKC-8 (*p* = 0.0017) cells was able to induce a migration advantage with a significantly higher percentage of wound closure when compared with the control condition (Figure 4A,C,D). Moreover, we also observed that the uptake of HCT 116-EVs condition 1 was able to induce a higher migration capacity in SK-HEP-1 in comparison to the internalization of HCT 116-EVs condition 2. Additionally, we also saw the effect of HCT 116-EVs condition 2 in HKC-8 cells, with an increase of their proliferation and migration capacity. Concerning HEPG-2 cells, we observed cell detachment in HCT 116-EVs condition 2, which did not allow the analysis of this experimental condition. Regarding the HCA-7 cells, after 48 h of the HCT 116-EVs’ incubation, an increase of migration capacity was observed, when compared to the control condition. 

The proliferation results are in agreement with the results observed for the migration capacity since we observed a higher proliferation rate in SK-HEP-1 (*p* < 0.001) after HCT 116-EVs condition 1’s uptake (Figure 4B). Additionally, there was also a significant increase of proliferation capacity of SK-HEP-1 (*p* = 0.003) and HKC-8 (*p* = 0.002) after stimulus with HCT 116-EVs 2. Thus, these results demonstrated that HCT 116-EVs promote cell migration capacity and proliferation of specific recipient cells, namely of SK-HEP-1 and HKC-8.

**Figure 5:**
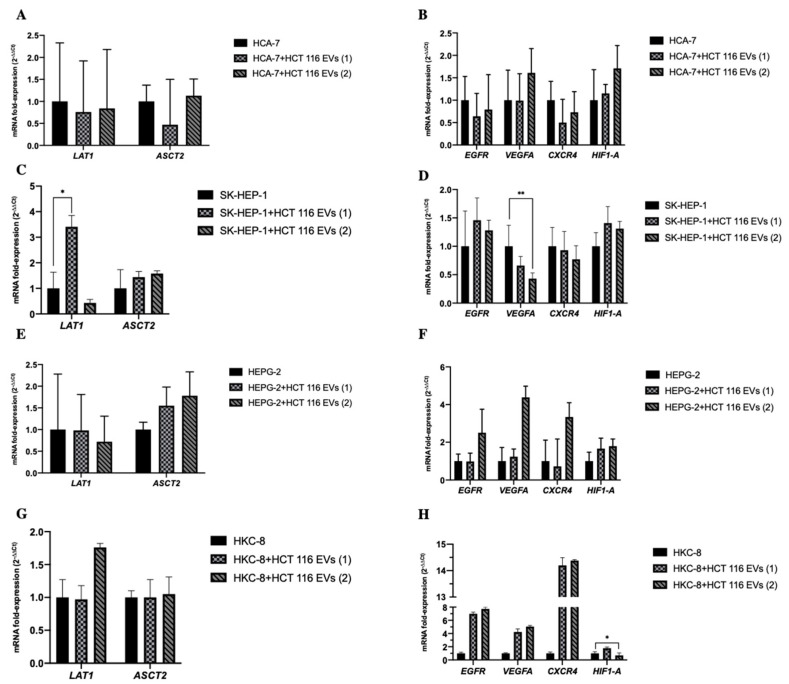
The bars represent the fold change of the mRNAs expression, normalized to *B2M*. Variation of levels of *LAT1*, *ASCT2*, *EGFR*, *VEGFA*, *CXCR4* and *HIF1-A* mRNAs in recipient cells after HCT 116-EVs’ uptake. Three biological replicates of each cell line were used for this experiment. (**A**,**C**,**E**,**G**) The graphs demonstrate that the HCT 116-EVs condition 1’s uptake leads to an increase of *LAT1* mRNA expression level by SK-HEP-1 cells. (**B**,**D**,**F**,**H**) Additionally, the HCT 116-EVs condition 2’s uptake decreases the expression level of *VEGFA* mRNA in SK-HEP-1 recipient cells. In HKC-8 recipient cells, the HCT 116-EVs condition 2 uptake induces a decrease of *HIF-1A* mRNA expression level. (Mean ± Std. Error, ** *p* < 0.001, * *p* < 0.05).

Considering the impact of the EVs’ cargo on recipient cells, we also analyzed the effect of HCT 116-EVs on the transcriptional profile of the different recipient cells (Figure 5). In SK-HEP-1 cell line, we observed an increase of *LAT1* (*p* = 0.049) mRNA levels after HCT 116-EVs 1’s uptake, and a decrease of *VEGFA* mRNA levels (*p* < 0.001) after HCT 116-EVs 2’ uptake (Figure 5B,F). Concerning the effect of the EVs’ uptake in the HKC-8 cell line (Figure 5D,H), we observed a decrease of *HIF1-A* (*p* = 0.019) mRNA levels after HCT 116-EVs 2’s uptake. Regarding the other cell lines, we did not find any statistically significant differences after HCT 116-EVs’ uptake.

**Figure 6:**
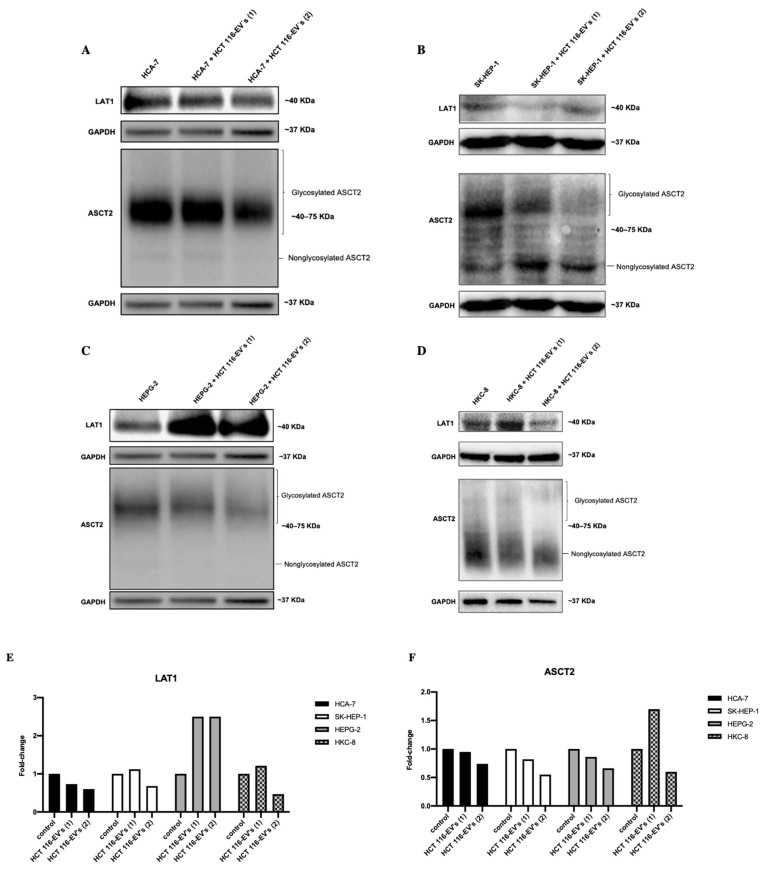
The experience was replicated two times for each sample. LAT1 and ASCT2 protein levels and HCT 116-EVs’ uptake effects in (**A**) HCA-7, (**B**) SK-HEP-1, (**C**) HEPG-2 and in (**D**) HKC-8 cell lines. Fold-change of the pixels’ volume adjusted intensity of (**E**) LAT1 and (**F**) ASCT2 protein levels, in recipient cells after HCT 116-EVs’ uptake. The bands show that HCT 116-EVs condition 1 internalized by SK-HEP-1 induce an increase of LAT1 protein level. Similarly, in HEPG-2 recipient cells, after HCT 116-EVs’ (condition 1 and 2) uptake, there is an increase of LAT1 protein levels. On the other hand, the internalization of HCT 116-EVs 1 by HKC-8 cells leads to an increase of ASCT2 protein levels.

Regarding the protein levels, the bands were quantified, as exemplified in Figure 6E,F. According to the results, we found a decrease of LAT1 and ASCT2’s protein level, when the HCA-7 cells were submitted to HCT 116-EVs’ (conditions 1 and 2) stimulus (Figure 6A,E). Concerning the SK-HEP-1 cells, we observed an increase of LAT1’s protein level after stimulus with HCT 116-EVs 1, compared to the control condition (Figure 6B,E). We also observed a change in the glycosylation status of ASCT2 after HCT 116-EVs’ (conditions 1 and 2) stimulus in SK-HEP-1 cells, which led to ASCT2’s protein expression in its non-glycosylated form, contrary to what happens in the control condition, where the ASCT2 protein is expressed in its glycosylated form. In HEPG-2 cells, we detected an increase of LAT1’s protein levels, and a decrease of ASCT2’s protein levels (Figure 6C,F). Additionally, we also found that in HCA-7 and HEPG-2, the glycosylation pattern of ASCT2 didn’t change after the EVs’ uptake, the ASCT2 protein being expressed mostly in its glycosylated form. In the HKC-8 cell line, we observed that the uptake of HCT 116-EVs (conditions 1 and 2) decreased LAT1’s protein levels compared to the control condition. Regarding ASCT2, we found an increased trend of the protein levels after HCT 116-EVs 1 stimulus. Furthermore, we observed that in the HKC-8’s basal condition and after HCT 116-EVs (1 and 2) stimulus, the ASCT2 expression is mostly expressed in its non-glycosylated form (Figure 6D,F). 

## 4. Discussion

One of the major concerns of CRC management is the fact that a high number of patients are diagnosed in advanced stages of disease, and that, in 50% of patients diagnosed with local diseases, metastasis eventually develops. In 30–50% of CRC patients, the liver is the predominant site of metastatic disease, in consequence of its drainage from the gastrointestinal tract [4,6,43,44]. In these cases, the only curative approach is surgery. However, a limited number of patients are considered eligible [44].

It is well established that the tumor microenvironment represents a complex network, in which tumor cells communicate with others cell types, including fibroblasts, endothelial cells and immune cells [45,46]. In recent years, the scientific community have focused on EVs’ intercellular communication role, and their potential as vehicles and mediators of cell communication and cellular microenvironment modulation has already been demonstrated [47,48]. Studies demonstrated that cancer derived EVs participate in critical steps of pre-metastatic niche formation in the primary tumor by delivering cargo to recipient cells in target organs [39,49]. The pivotal role of EVs in regulating several immune-related pathways leading to activation, differentiation and expression of immune cells and modulation of the tumor microenvironment has already been demonstrated, as well as its significant role in CRC progression and metastasis [50]. Increasing evidence shows that mRNAs can be transferred to the surrounding microenvironment, via EVs’ pathways, and influence the metabolism of recipient cells to favor cancer progression [49,51]. In fact, according to Chiba and colleagues, EVs derived from three CRC cells (HCT-15, SW480 and WiDr) showed the capacity to transfer mRNAs into 2D A549 cells (lung cancer cells) and HEPG-2 cell lines, validating that EVs-derived RNAs can be shuttled between cells, and can be involved in the regulation of gene expression in recipient cells [40]. Furthermore, Shao and co-workers described that CRC derived EVs present a pivotal role in promoting liver metastasis, by inducing a premetastatic niche through miR-21-TLR7-IL-6 axis [43]. These authors described that CRC-EVs can specifically target liver tissue and induce liver macrophages toward an IL-6 proinflammatory phenotype [43]. Additionally, the study mentions miR-21 as highly enriched in CRC-EVs, this miRNA being essential for creating a proinflammatory phenotype in the liver and creating liver metastasis in CRC [43]. Finally, the authors also demonstrate that silencing either miR-21 in CRC-EVs, or TLR-7 in macrophages, abolished the CRC-EVs’ induction of proinflammatory macrophages [43]. Costa-Silva and co-workers described, for the first time, the sequential steps responsible for the formation of liver pre-metastatic niches (LPMN) supportive of PC metastasis, which involved binding of Macrophage Migration inhibitory factor (MIF)+ PC-derived EVs to liver Kupffer cells, followed by TGF-β production by these cells. TGF-β, in turn, promoted fibronectin production by hepatic stellate cells, that supported the accumulation of bone marrow-derived macrophages, completing the LPMN formation [52]. More recently, Xuan and colleagues reported that EVs derived from breast cancer contribute to pre-metastatic niche formation and promote bone metastasis of tumor cells [53]. This process is mediated by EVs derived from breast cancer cells (SCP28 and MDA-MB-231 cells), which promote osteoclast differentiation and enhance bone metastasis [53]. Therefore, these studies suggest the role of EVs in metastasis establishment, through the transfer of biomolecular cargo, presenting a great potential to be used as predictive targets. 

Nevertheless, there is still no evidence in the literature about the influence of *LAT1* and *ASCT2* mRNAs derived from CRC-EVs in CRC progression and metastasis, even though they play an important role in the growth and survival of CRC cancer cell lines, since they ensure the rapid exchange of AA and the maintenance of an AA pool in the cytosol [54]. In this study, we detected for the first time the presence of *LAT1*, *ASCT2* and other oncogenic mRNAs on CRC-EVs, as well as their capacity to modify the transcriptional profile and phenotypic characteristics of recipient cell lines. Moreover, we showed the presence of *EGFR*, *VEGFA*, *CXCR4* and *HIF1-A* mRNAs in CRC-EVs, which is in agreement with several evidences that support the involvement of these molecules in CRC development and metastases formation [26,55,56,57,58,59,60,61,62,63,64]. The overexpression of EGFR, HIF-1α and VEGFA has been described in CRC, being associated with poor prognosis, aggressiveness, and a higher potential of metastases formation [59,62,65]. Moreover, CXCR4 is also correlated with poor histological differentiation, distant metastasis and lymph node metastasis, being its higher levels associated with poor prognosis in CRC patients [66]. 

Thus, we hypothesize that the incorporation of these molecules on EVs are essential to trigger the establishment of a microenvironment that supports the metastases’ formation. In fact, we observed an increase of *LAT1* and a decrease of *VEGFA* mRNAs in SK-HEP-1, and an increase of *HIF1-A* mRNA levels in HKC-8 cells after stimulus with HCT 116-EVs. These molecular changes may be associated with the release of pro-inflammatory cytokines such as IL-6, leading to an inflammatory process which is influenced by cellular metabolism and hypoxia [67]. In fact, Quan and colleagues, showed that LAT1 was required for angiogenic processes, since VEGFA’s stimulus induced LAT1 overexpression that consequently triggered angiogenesis [68]. On the other hand, Shi and co-workers describe that, in non-small cell lung cancer, the expression of LAT1 is correlated with HIF1-A levels [69,70]. Therefore, according to our results, we can assume that an increase in *HIF-1A* and hypoxia leads to an increase of *LAT1* expression [70]. Interestingly, the increase of *LAT1* mRNA in the SK-HEP-1 cell line, after HCT 116-EVs’ uptake, was able to support the high demand of amino acids by these cells. In fact, the increase of *LAT1* mRNA in SK-HEP-1 translated into the increase of LAT1 protein expression, cell proliferation, as well as in the invasion capacity of these cells. Wang and co-workers had already reported higher protein levels of CXCR4 in the liver of the HT-29-derived exosome-treated Caco-2-implanted mice [71]. 

Interestingly, we also observed that the internalization of different concentrations of HCT 116-EVs cause different effects in recipient cells. In fact, we observed that SK-HEP-1 cells reach a saturation peak after HCT 116-EVs condition 1’s stimulus for around 24 h. Additionally, we also saw that there is a dose dependency of EVs administrated to SK-HEP-1 recipient cells, since the migration and proliferation capacity displayed a strong dose dependence with a minimal effective dose of 5.3 × 10^8^ EVs/mL (HCT 116-EVs condition 1). Similarly, Franzen and colleagues also demonstrated that the EVs’ uptake by recipient cells is time and dose dependent, a peak of EVs’ internalization occurring before the 24 h of incubation [72]. These authors described that the recipient cells reached a saturation point of exosomes internalization after 14 h, however, after 24 h of stimulus the authors demonstrated that exosomes continued to be taken up by cells [72]. Similarly, Jurgielewicz and co-workers also describe that HEK293T-EVs’ uptake is time and dose dependent, the peak of uptake being around 12 h [73]. Moreover, the authors also report that after a dose of 6000 EVs/cell are taken up by HEK293T recipient cells, these reach a dose saturation limit [73]. Jurgielewicz and co-workers also report that since EVs have shown to be internalized then released after 24 h, longer incubations may generate inaccurate internalizations readouts [73]. In fact, after 48 h of incubation with HCT 116-EVs condition 2, the results of migration and proliferation assays in HKC-8 cells seem to be inconsistent, which could be consequence of the long EVs’ incubation period, and could be associated in an inaccurate internalization readout. 

Considering the key role of LAT1 during CRC progression, and specially the capacity of tumor cells to encapsulate mRNA molecules inside EVs to modulate the surrounding microenvironment, the development of pharmacological strategies based on the inhibition of LAT1 could be promising for CRC patients’ management. In fact, Okano and co-workers have already reported that JPH203, a LAT1 inhibitor, demonstrated potential to be used for CRC patients’ treatment [23]. In a phase I study, JPH203 treatment was well tolerated by patients and led to disease control in two of the six CRC patients and in three of the five patients with biliary tract cancer [23]. 

The upregulation of *LAT1* mRNA could also be associated with changes in the ASCT2 glycosylation pattern. In fact, a study performed by Polet and colleagues reports that glucose availability regulates the glycosylation of ASCT2 [74]. The authors describe that inhibition of glucose metabolism prevents ASCT2 glycosylation and promotes LAT1 upregulation as a countertrading mechanism of glycosylation’s inhibition [74]. We observed a change in the ASCT2 glycosylation pattern after HCT116-EVs stimulus. The changes previously described in SK-HEP-1 were also followed by changes in ASCT2 protein’s conformation, that changed to a non-glycosylated form. This may be due to the fact that HCT 116-EVs stimulate metabolic deregulation of SK-HEP-1 cells, which could lead to an increase of glucose consumption by these cells, and consequently, lead to a change of the glycosylation status of the ASCT2 protein. 

In conclusion, the present study supports the role of CRC-EVs as key mediators of tumoral progression, supporting a proangiogenic and proliferative microenvironment establishment. 

## 5. Conclusions

Our results uncovered an additional role of EVs in aggressive phenotypes of CRC, through the transference of *LAT1* mRNA, with a phenotypic impact on cell proliferation and invasion capacity. Moreover, future studies should consider the replication of this in vitro study in a three-dimensional (3D) cell culture model to validate the cellular response to HCT 116-EVs’ stimulus. These models provide more physiologically information and predictive data for in vivo tests since they mimic the biological conditions. In 3D cell culture models, cancer cells can maintain the shape, polarity and the heterogeneity observed in vivo. On the other hand, after the cell culture validation it will be crucial to check the influence of the HCT 116-EVs in animal models, to elucidate the role of this structure in the metastasis formation and clarify the metastatic routes of CRC. One possible approach to validate the hypothesis raised in this study may be to study if the inoculation of HCT 116-EVs only presents tropism for liver and kidney cells, or if they are able to affect the proliferation and migration capacity of cells from other organs. Additionally, it would also be interesting to validate our findings in CRC patients’ plasma EVs to evaluate the biomarker potential of CRC EVs-derived mRNA, especially *LAT1*, in patients’ prognoses and follow-ups. The validation of the biomarker potential of CRC EVs-derived mRNAs would be useful for liquid biopsies’ implementation and the development of new therapeutic approaches for CRC. 

## Data Availability

Not applicable.

## Supplementary Materials

The following are available online at https://www.mdpi.com/article/10.3390/biology11010145/s1, Figure S1: Representative images of wound healing assay in (A) HCA-7, (B) SK-HEP-1, (C) HEPG-2 and (D) HKC-8 recipient cells, with respective uptakes of HCT 116-EVs (1 and 2), in the different time points. Figure S2: Representative original images of Western Blot assay in (**A**) HCA-7, HEPG-2, (**B**) SK-HEP-1 and (**C**) HKC-8 recipient cells, with respective uptakes of HCT 116-EVs (1 and 2).
