# Supplementary material for: Extracellular Vesicles Derived-LAT1 mRNA as a Powerful Inducer of Colorectal Cancer Aggressive Phenotype
Source: Biology (Basel). 2022 Jan 15;11(1):145. doi: 10.3390/biology11010145 (PMC8773288; doi:10.3390/biology11010145)
Supplement: Supplementary file 1 [file biology-11-00145-s001.zip › biology-1525095-supplementary.pdf]

Supplementary Material

Extracellular vesicles derived-LAT1 mRNA as a powerful inducer of colorectal cancer aggressive phenotype

Cristina Almeida [1,*], Ana Luísa Teixeira [1,3,*], Francisca Dias[1], Vera Machado [1], Mariana Morais [1], Gabriela Martins [4], Carlos Palmeira [4,5,6], Sílvia Batista [7], Bruno Costa-Silva [7] and Rui Medeiros [1,2,3,6,7,8]

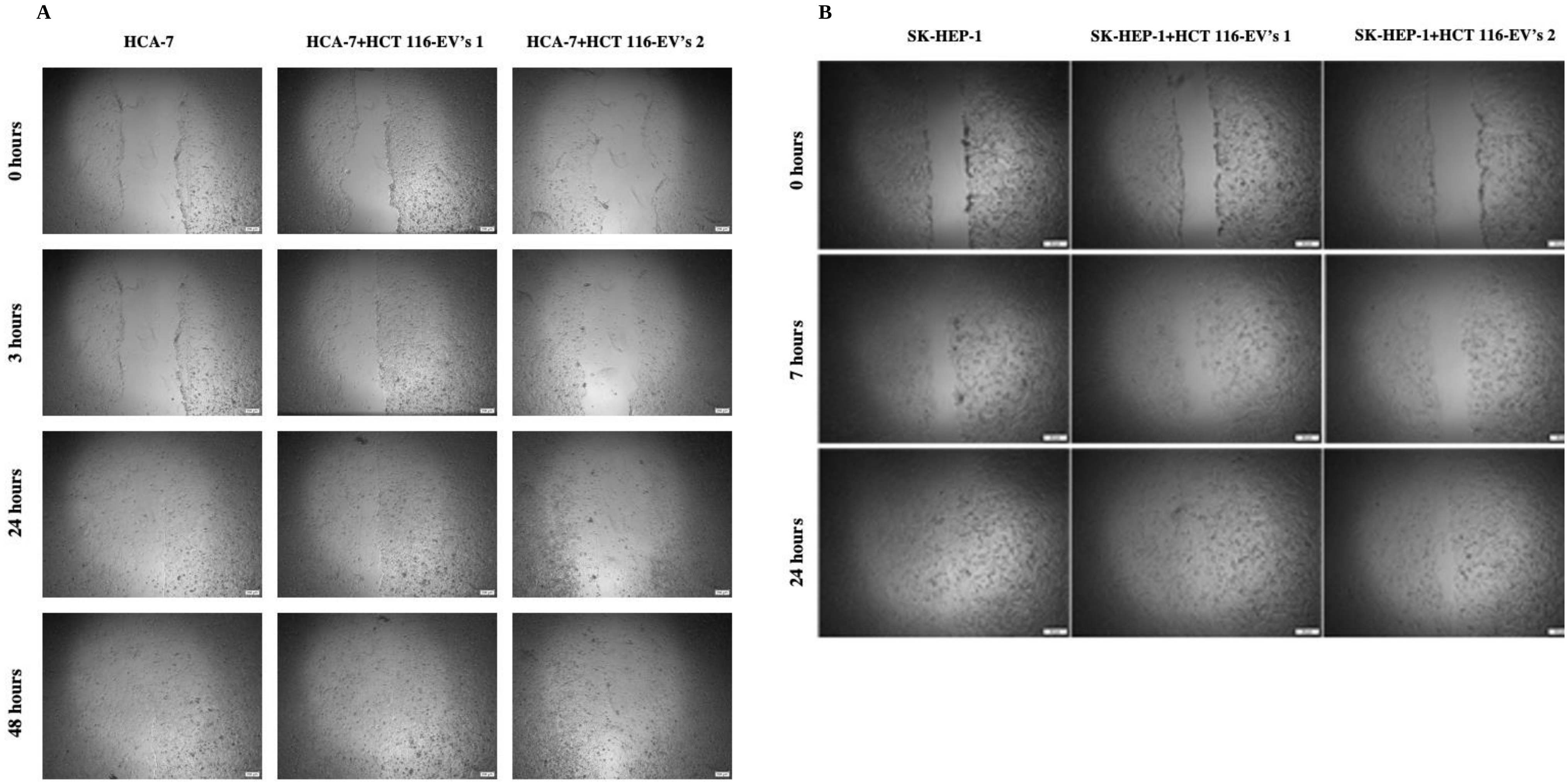
A
HCA-7
HCA-7+HCT 116-EV's 1
HCA-7+HCT 116-EV's 2
0 hours
3 hours
24 hours
48 hours
B
SK-HEP-1
SK-HEP-1+HCT 116-EV's 1
SK-HEP-1+HCT 116-EV's 2
0 hours
7 hours
24 hours

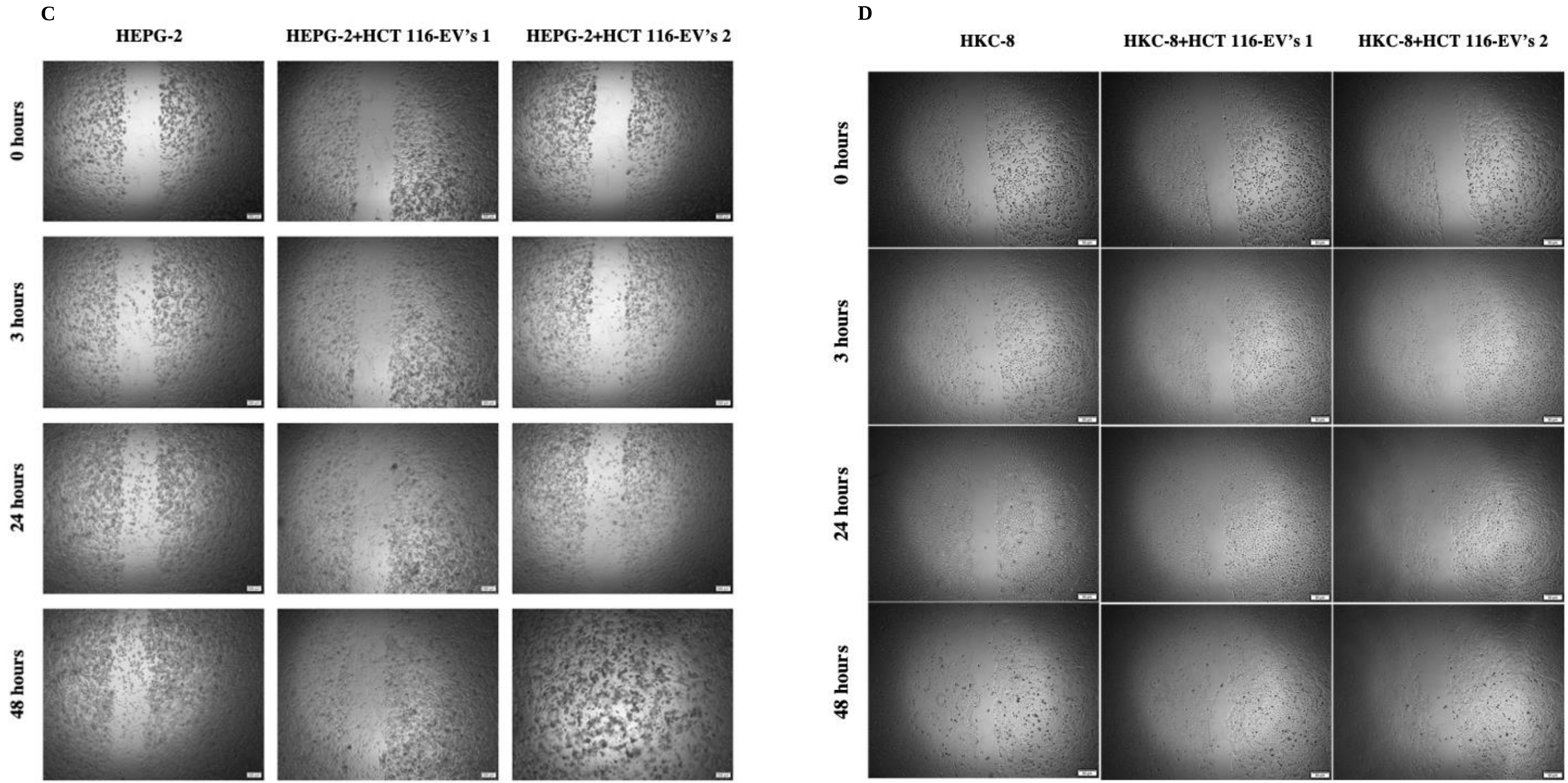

Figure Supplementary 1. Representative images of wound healing assay in (**A**) HCA-7, (**B**) SK-HEP-1, (**C**) HEPG-2 and (**D**) HKC-8 recipient cells, with respective uptakes of HCT 116-EV's (1 and 2), in the different time points. (**A**) and (**B**) Scale bar = 200 μm.

A

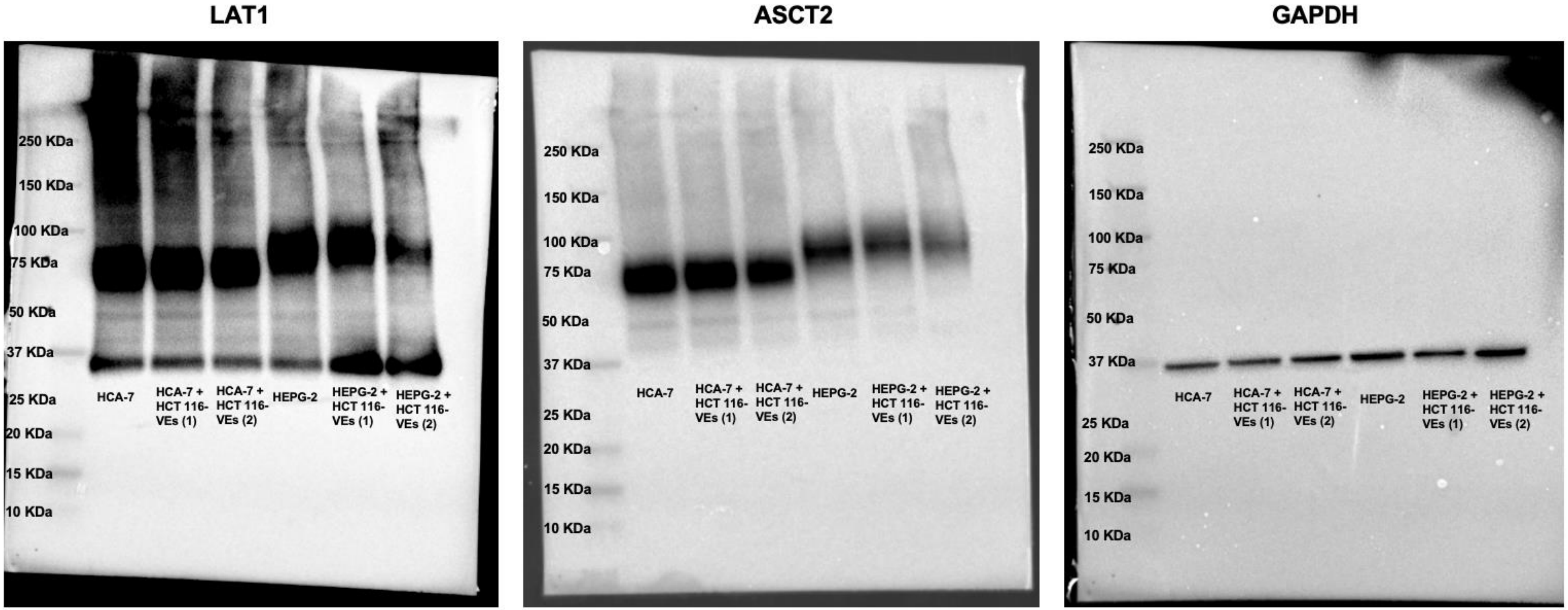
LAT1
ASCT2
GAPDH
250 KDa
150 KDa
100 KDa
75 KDa
50 KDa
37 KDa
25 KDa
20 KDa
15 KDa
10 KDa
HCA-7
HCA-7 + HCT 116-VEs (1)
HCA-7 + HCT 116-VEs (2)
HEPG-2
HEPG-2 + HCT 116-VEs (1)
HEPG-2 + HCT 116-VEs (2)

B

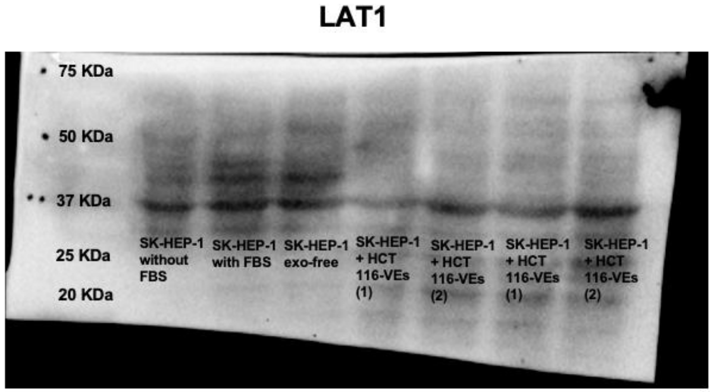
LAT1
75 KDa
50 KDa
37 KDa
25 KDa
20 KDa
SK-HEP-1 without FBS
SK-HEP-1 with FBS
SK-HEP-1 exo-free
SK-HEP-1 + HCT 116-VEs (1)
SK-HEP-1 + HCT 116-VEs (2)
SK-HEP-1 + HCT 116-VEs (1)
SK-HEP-1 + HCT 116-VEs (2)

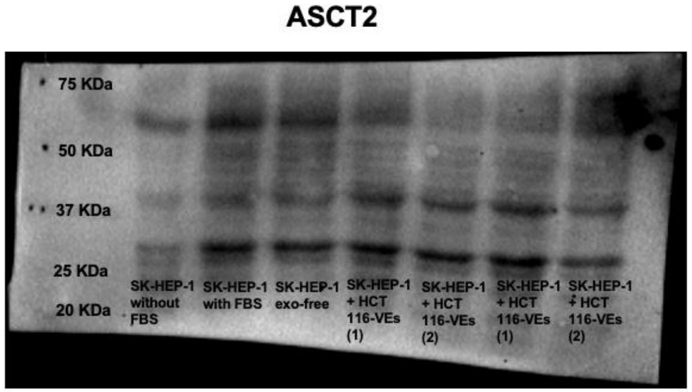
ASCT2
75 KDa
50 KDa
37 KDa
25 KDa
20 KDa
SK-HEP-1 without FBS
SK-HEP-1 with FBS
SK-HEP-1 exo-free
SK-HEP-1 + HCT 116-VEs (1)
SK-HEP-1 + HCT 116-VEs (2)
SK-HEP-1 + HCT 116-VEs (1)
SK-HEP-1 + HCT 116-VEs (2)

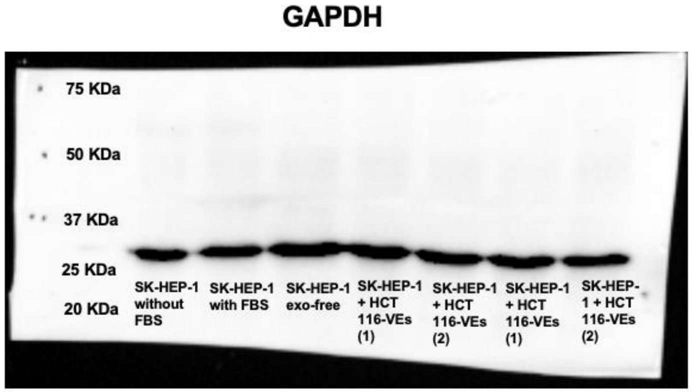
GAPDH
75 KDa
50 KDa
37 KDa
25 KDa
20 KDa
SK-HEP-1 without FBS
SK-HEP-1 with FBS
SK-HEP-1 exo-free
SK-HEP-1 + HCT 116-VEs (1)
SK-HEP-1 + HCT 116-VEs (2)
SK-HEP-1 + HCT 116-VEs (1)
SK-HEP-1 + HCT 116-VEs (2)

C

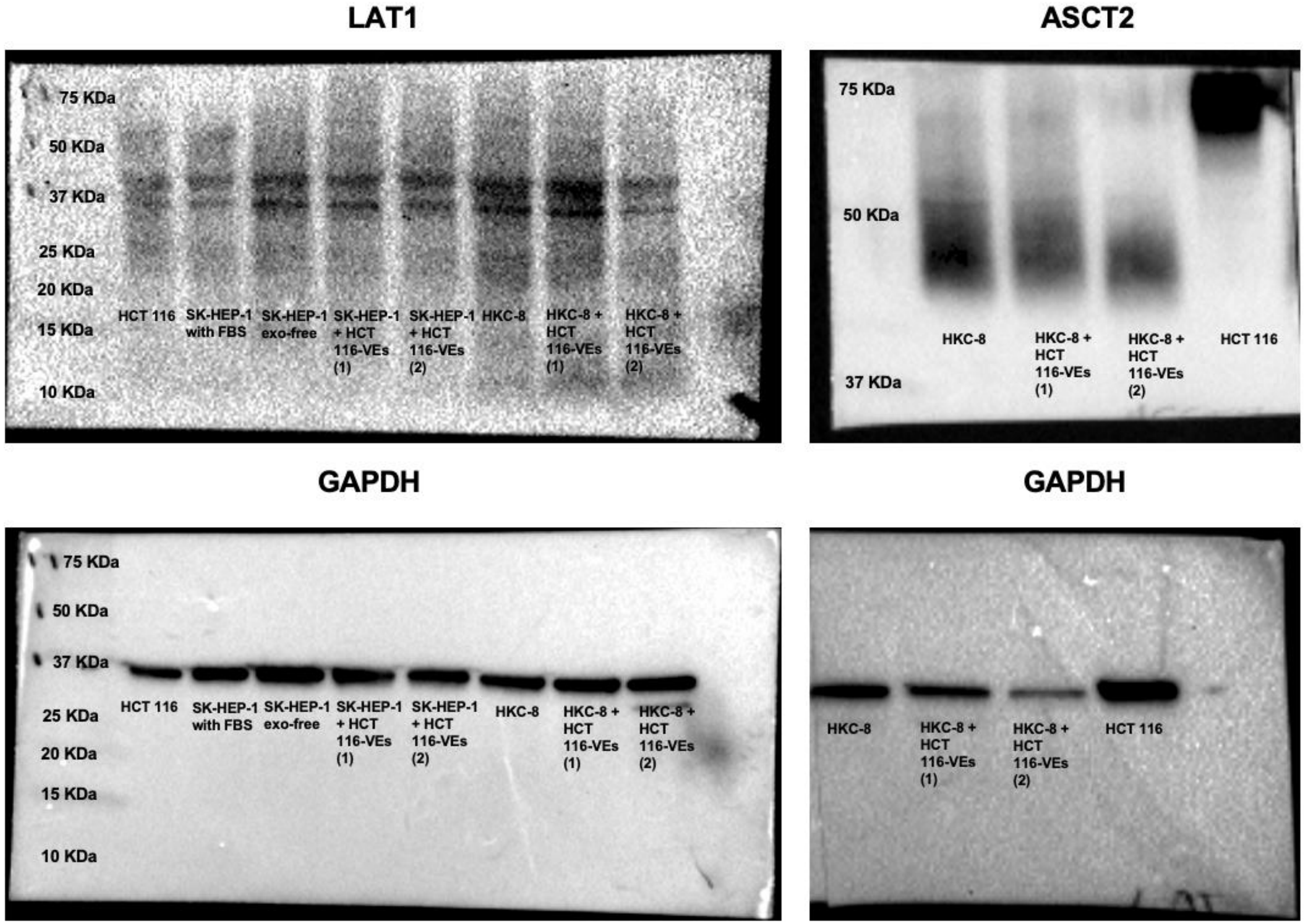

Figure Supplementary 2. Representative original images of Western Blot assay in **A)** HCA-7, HEPG-2, **B)** SK-HEP-1 and **C)** HKC-8 recipient cells, with respective uptakes of HCT 116-EV's (1 and 2).
